# MRI and single-cell RNA sequence results reveal the influence of anterior talofibular ligament injury on osteochondral lesions of the talus

**DOI:** 10.1186/s13018-024-04826-5

**Published:** 2024-08-10

**Authors:** Jie Xu, Siyi Yang, Ruiqi Fan, Hongbo Wu, Hui Mo

**Affiliations:** 1 Faculty of Chinese Medicine and State Key Laboratory of Quality Research in Chinese Medicine, Macau University of Science and Technology, Macau, 999078 China; 2 Macau University of Science and Technology, Macau, 999078 China; 3 School of Clinical Medicine, Beijing University of Chinese Medicine, Beijing, 100000 China; 4 The First School of Clinical Medicine, Nanjing University of Chinese Medicine, Nanjing, 210023 China

## Abstract

Anterior talofibular ligament injuries and osteochondral lesions of the talus present unique challenges to orthopedic surgeons. This study aimed to investigate the relevant relationship between them by analyzing the Magnetic resonance imaging (MRI) results of clinical patients and single-cell RNA sequence (scRNA seq) results of healthy talus cartilage to discuss the risk factors. Data from 164 patients from 2018 to 2023 was retrospectively analyzed. The correlation analysis between ATFL injury grade and the Hepple stage of OLT determined by MRI was performed. Publicly available single-cell RNA datasets were collected. Single-cell RNA datasets from five volunteers of healthy talus cartilage were analyzed. ATFL injury grade was relevant with the Hepple stage of OLT (*P* < 0.05). The results of multivariate logistic regression analysis showed that injured area was the independent influencing factor of the incidence rate and the severity of OLT (*P* < 0.05). The Hepple stage of OLT was relevant with AOFAS and VAS (*P* < 0.05). Single-cell RNA sequence results showed that among the 9 subtypes of chondrocytes, the interaction strength between HTC-A and HTC-B is the highest. Their physical interactions are mainly achieved through the CD99 signaling pathway, and factor interactions are mainly achieved through the ANGPTL signaling pathway. Anterior talofibular ligament injury may lead to osteochondral lesions of the talus. Early medical intervention should be carried out for ligament injuries to restore joint stability and avoid cartilage damage.

## Introduction

The ankle is the main weight-bearing joint [1]. Ankle sprain is a high-incidence sports injury, accounting for about 40% of it [2, 3]. The ankle joint is prone to varus injury, which leads to the tear of the lateral ankle ligament. Among these ligaments, the anterior talofibular ligament (ATFL) is the most frequently injured one [4]. Studies have shown that among all ankle joint ligament injuries, ATFL accounts for as high as 66% [5, 6]. The ankle joint typical symptoms are persistent stiffness, swelling as well as pain, which may be accompanied by secondary synovitis, tendinitis and muscle weakness. ATFL injury remains one of the research highlights.

Osteochondral lesions of the talus (OLT) are common in clinics, of which trauma is the main pathogenic factor [7]. It accounts for more than half of acute ankle joint injuries. OLT mainly refers to the local articular cartilage peeling, which may also implicate the deep subchondral bone [8, 9]. The common symptoms are joint pain, fluid swelling, and joint dysfunction, which even lead to disability in severe cases. Study on the OLT is of great significance.

MRI is characterized by high resolution in the examination of soft tissue as well as multi-parametric and multi-planar imaging [10]. It has obvious advantages in displaying complex structures and tissue layers of ligaments and has good diagnostic value for early diagnosis of ATFL injuries [11–13]. Although ordinary plain radiography films and conventional CT examinations are commonly used in bone diseases, it is difficult to display cartilage, especially whether small bone fragments of cartilage are displaced [11]. Ankle arthroscopy can clearly show the degree of cartilage lesions, which can be used as a more accurate and effective means to diagnose OLT, but as a traumatic examination, it is not widely accepted [14, 15]. At present, MRI joint examination technology is relatively mature, which can not only show bone lesions, but also better show cartilage damage [16]. It has become the first choice for preoperative evaluation of articular cartilage damage [17, 18]. MRI results of ATFL injury and OLT may reveal some correlations between them.

Single-cell RNA Sequence has been developed well in recent years. Compared to traditional methods of profiling bulk populations of cells, this technique provides us with additional dimensions for studying the correlation between diseases and the interactions between cells [19]. Single-cell RNA Sequence allows for objective, high-throughput and high-resolution single-cell analysis [20].

Interestingly, we found that in the clinic most patients with ATFL injuries were accompanied by OLT. However, in previous studies, there was no article about the relationship between them. Therefore, this study investigate the relevant relationship between ATFL injury and OLT by analyzing the MRI results of clinical patients and single-cell RNA sequence results of healthy talus cartilage obtained from 5 volunteers, and to discuss the risk factors.

## Results

### Correlation analysis of ATFL injuries and OLT

The results of correlation analysis showed that ATFL injury grade was relevant with the Hepple stage of OLT (Table 1). This proves that ATFL injury may lead to OLT.

**Table 1:** Correlation analysis of ATFL injuries and OLT

Variable	Hepple stage of OLT	
	r	*P*
ATFL injury grade	0.244	0.040^*^

*OLT* Osteochondral lesions of the talus; *ATFL* Anterior talofibular ligament

*P < 0.05

## Severity and risk factors *of talus* cartilage under different factors

The description of grouping and assignment of factors are showed in Table 2. The results of multivariate logistic regression analysis showed that injured area was the independent influencing factor of the incidence rate and the severity of OLT (*P* < 0.05, Table 3). While gender, age, course of disease, injured side, and thickness index was not the independent influencing factor of the incidence rate and the severity of OLT (*P* > 0.05, Table 3).

**Table 2:** Description of grouping and assignment of factors

Variable	Grouping and assignment
*Dependent variable*	
Hepple classification	Stage I = 1, Stage IIa = 2, Stage III = 3, Stage IV = 4, Stage V = 5, Normal = 6
*Independent variable*	
Gender	Male = 0, Female = 1
Age	Adolescence = 0, Maturity = 1
Course of disease	Acute stage = 0, Chronic stage = 1
Injured side	Left = 0, Right = 1
Injured area	Small = 0, Large = 1
Thickness index	Continuity variable

**Table 3:** Comparison of damage degree of talus cartilage under different factors

Influence factor	Number of cases	Normal	Stage I	Stage IIa	Stage IV	Stage V	X^2^	*P*
*Gender*								
Male	40	16	8	10	5	1	2.515	0.642
Female	31	17	8	3	2	1		
Age							1.051	0.902
Adolescence	41	19	9	8	4	1		
Maturity	30	14	7	5	3	1		
*Course of disease*								
Acute stage	59	28	11	8	6	2	4.452	0.348
Chronic stage	12	2	3	4	0	0		
*Injured side*								
Left	36	20	5	8	1	2	9.276	0.055
Right	35	13	11	5	6	0		
Injured area							87.104	0.000^*^
Small	34	33	1	0	0	0		
Large	37	0	15	13	7	2		
Thickness index	71						1.167	0.883

*P < 0.05

## Correlation analysis of ATFL injury grade and the Hepple stage of OLT and AOFAS and VAS

The correlation analysis of ATFL injury grade and the Hepple stage of OLT and AOFAS and VAS showed that the Hepple stage of OLT was relevant with AOFAS and VAS (*P* < 0.05, Table 4).

**Table 4:** Correlation analysis of ATFL injury grade and the Hepple stage of OLT and AOFAS and VAS

Variable	AOFAS		VAS	
	r	*P*	r	*P*
Hepple stage of OLT	0.596	0.000^*^	0.430	0.000^*^
ATFL injury grade	-0.183	0.127	− 0.113	0.348

*OLT* Osteochondral lesions of the talus; *ATFL* Anterior talofibular ligament; *AOFAS* American orthopaedic foot and ankle association; *VAS* Visual analog scoring

*P < 0.05

## Classification and interaction of chondrocytes in the talus

**Fig. 1:**
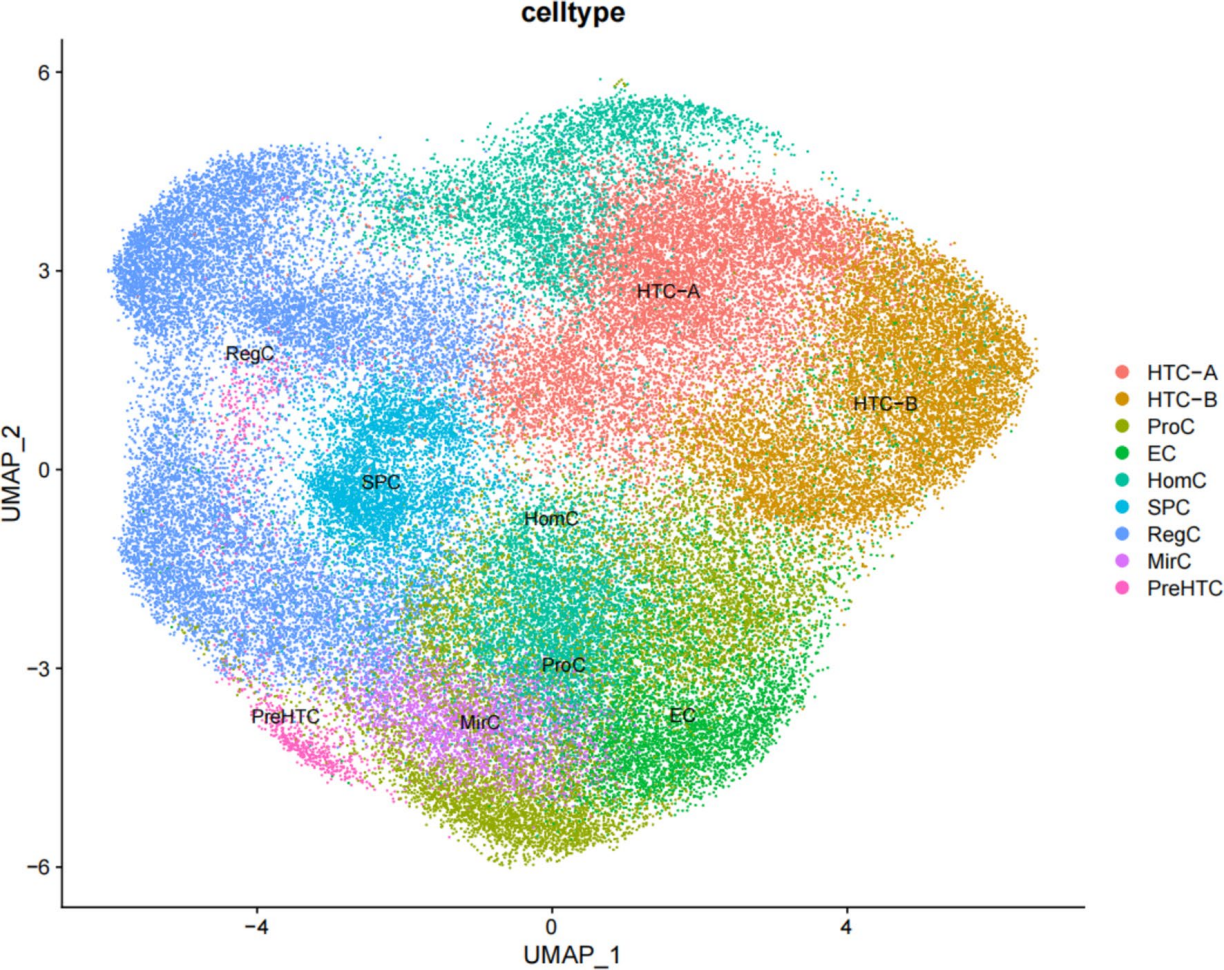
Result for cell-type annotation

**Fig. 2:**
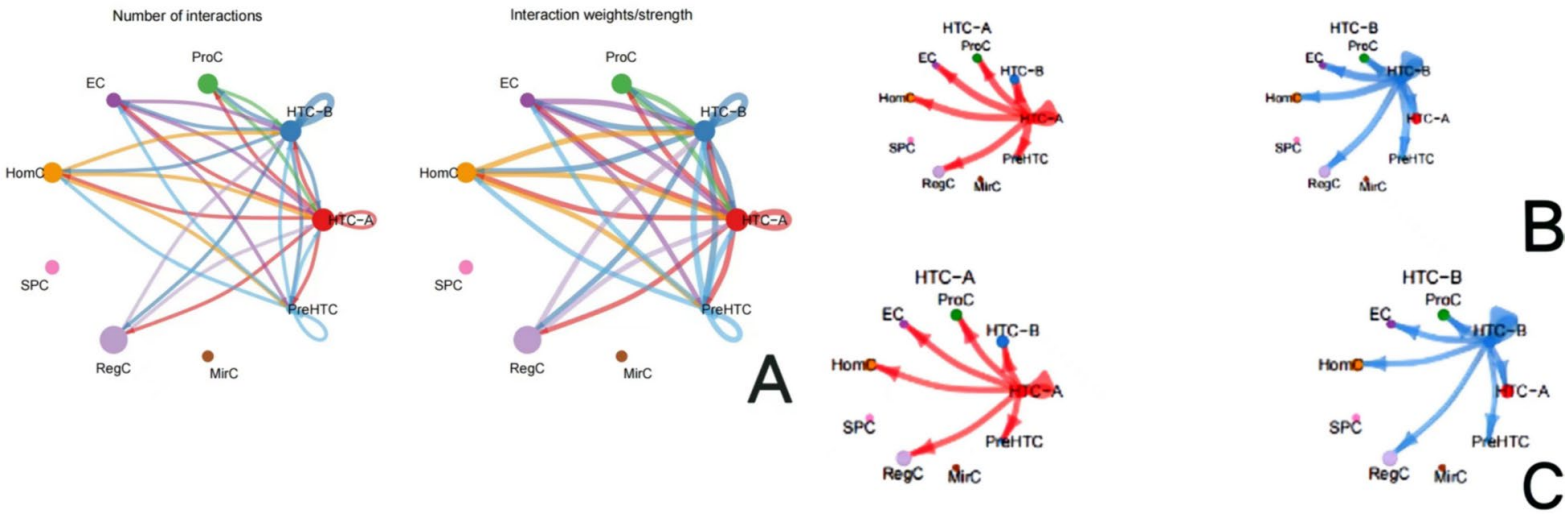
Results for the cell–cell interaction among cell types. **A** Interaction number and weights/strength for cell–cell communication among each cell type. The size of the circle represents the ratio of each cell type; the width of the line represents the strength of cell–cell communication. **B** Interaction number for cell–cell communication among HTC-A with other cell types and HTC-B with other cell types. **C** Interaction weights/strength for cell–cell communication among HTC-A with other cell types and HTC-B with other cell types

**Fig. 3:**
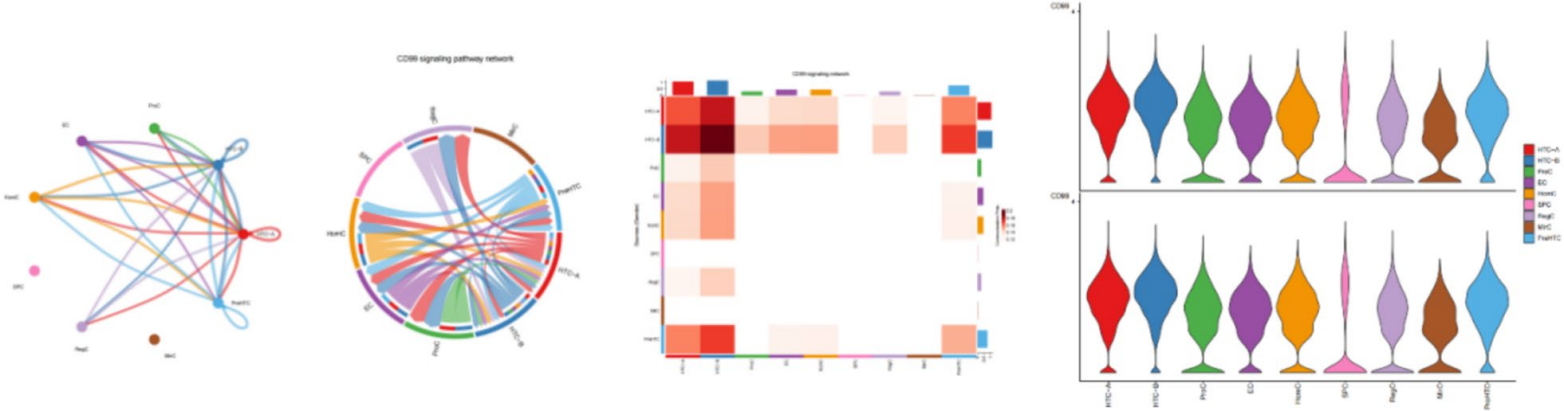
The communication network between cells (CD99 signaling pathway)

**Fig. 4:**
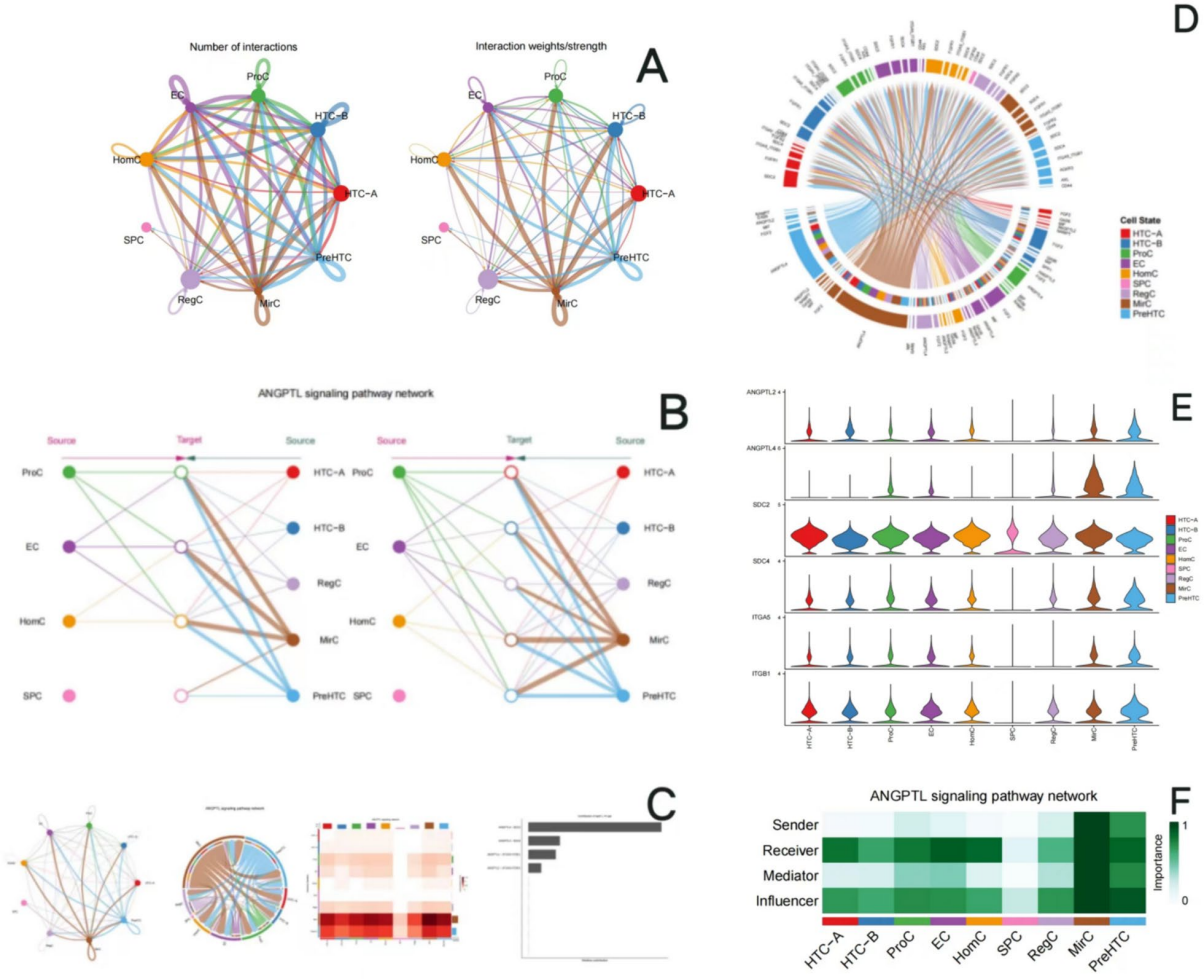
Results for the factor interactions among cell types. **A** Interaction number and weights/strength for factor interactions among each cell type. The size of the circle represents the ratio of each cell type; the width of the line represents the strength of cell–cell communication. **B**, **C** The communication network between cells (ANGPTL signaling pathway). **D**, **E** Display of the expression of signaling genes related to important communication of signaling pathways and inference in cell populations (ANGPTL signaling pathway). **F** Heatmap for the role of each cell type in different pathways; the color of the rectangle represents the extent of importance in each role

Clustering and cell-type annotation results are shown in Fig. 1. Single-cell RNA sequence results showed that among the 9 subtypes of chondrocytes, the interaction strength between HTC-A and HTC-B is the highest. Figure 2 shows the results for the cell–cell interaction among cell types. The size of the circle and the width of the line represent the ratio of each cell type and the strength of cell–cell communication, respectively. Their physical interactions are mainly achieved through the CD99 signaling pathway (Fig. 3), and factor interactions are mainly achieved through the ANGPTL signaling pathway (Fig. 4).

## Discussion

Lateral ligament injury of the ankle joint is common. The ATFL is important for ankle joint stability but it is weaker than other lateral ligaments [21, 22]. It is the first and most easily damaged in ankle varus [23, 24].

Approximately 57% of the surface of the talus is covered by articular cartilage [6]. Among them, the largest joint cartilage has no vascular distribution inside the talus trochlear. It lacks self-healing ability when damaged, and in clinical practice, it usually manifests as persistent ankle pain [25, 26]. As the injury gradually worsens, it will affect the upper surface of the talus, causing greater and irreversible damage to the entire ankle joint [27–29].

There is a research report that by collecting CT and MRI data of patients with ATFL rupture and 2-month follow-up after Broström surgery, by establishing finite element model of the foot and ankle to simulate the stress on the ankle joint of patients with ATFL injury before and after surgery during normal gait cycles (landing phase, neutral phase, off ground phase) [30]. Furthermore, the stress distribution and maximum stress value of the talus cartilage can be measured, and then their differences can be analyzed. The conclusion of this research is that the ATFL has a certain protective effect on the talus trochlear regardless of the phase. Under the action of the ATFL, the stress situation of the talus trochlear has been improved to a certain extent, it is indispensable for the ankle stability [31]. What's more, the maximum stress values all occur on the surface of the talus trochlear near the inner side of the ankle joint. The influence of the ATFL on the talus trochlear during the off-ground phase is much greater than that during the landing and neutral phases. This indicates that under certain circumstances, the greater the torque on the ankle joint, the more significant the effect of the ATFL on the stability of the talus trochlear [30]. The limitation of this study is that there may still be significant differences between the modeling analysis and the actual ankle structure and function, but it provides a theoretical basis for the research of this article.

In our study, the etrospectively correlation analysis showed that ATFL injury grade was relevant with the Hepple stage of OLT. This is the analyzed results of 164 patients’ data from our hospital. This supplements the lack of existing literature and proves ATFL injury may lead to OLT. We believe that when the ATFL is injured, the talus may undergo inversion and forward movement during ankle movement, resulting in ankle subluxation [32]. The medial side of the talus crest may collide with the malleolus crest, causing wear and tear of the medial cartilage of the talus crest [33]. This inspires us ankle arthroscopy and ligament repair have effective treatment for the anterior lateral impact of the ankle joint, alleviate pain, enhance ankle stability, and effectively protect ankle cartilage [34, 35].

What’s more, the results showed that injured area was the independent influencing factor of the incidence rate and the severity of OLT, while gender, age, course of disease, injured side, and thickness index was not. This indicates that the area of injury may serve as an important observational indicator for determining the severity and prognosis of the disease in clinic. This study also verified that the Hepple stage of OLT was relevant with AOFAS and VAS.

In addition, there are two sources of OLT: one is primary injury, and the other is caused by ATFL injury, including at the same time of it and after the injury because of joint instability [36]. This study is mainly to understand OLT related to ATFL injury. However, the patient older are easier to get cartilage injuries. To minimize the interference of confounding factors, when formulating the inclusion criteria, patients over 40 years old and those with ankle disease, deformity, and recurrent ankle pain before suffering from ATFL injury were excluded. Because the bone structure of juvenile patients is not completely developed, which is quite different from that of adult patients, patients under 18 years old are also excluded [37–39].

Therefore, the population selected for this study is 18–40 years old. This population has high activity intensity and high exercise requirements [40]. It is a group with a high incidence of ATFL injury and the most active group receiving the medical intervention. The cartilage damage observed in young people without ankle malformation and trauma history is less affected by factors such as old age. All in all, It can be considered that OLT of patients in this study occurs at the same time of ATFL injury or after the fracture, which means it is a concomitant change of ATFL injury.

Furthermore, besides the macro level impact analysed above, is there also an interaction between the ATFL injury and OLT at the micro level? For this purpose, single-cell sequencing data were selected and analyzed in this study for further analysis [41, 42].

Publicly available single-cell RNA datasets were collected and analyzed in this article. The typing of talus chondrocytes were validated, the interaction between cells were studied, and the related pathways of interaction were analyzed. The results provide foundation for the pathogenesis and treatment of OLT. This suggests that intercellular interactions may be the reason why ATFL may lead to OLT.

Lateral ankle sprains account for 85% of all ankle sprains, and most commonly occur in sports activities. ATFL is the weakest and most frequently injured in the lateral ligament complex of the ankle joint [43–45]. OLT is usually associated with ankle joint pain and dysfunction. They can occur after ankle trauma. To summarize, this study investigated the influence of ATFL on OLT, discussed the risk factors that lead to the occurrence of OLT, and analyzed the interactions between talus chondrocytes. The work of this study suggests early medical intervention for ligament injuries to avoid secondary cartilage damage. These research results will be beneficial for clinical examination, diagnosis, and treatment [46–53].

There are a few limitations to this study. Due to a lack of data on single-cell sequencing of chondrocytes in the talus, only 5 healthy samples were found and included in our research. The sample size was too small. As a result, this study did not compare the control group with the disease group. In the future, Single-cell sequencing research on ATFL injuries and OLT is needed to compensate for the shortcomings of this article.

## Methods

### Volunteers

This study was approved by the Ethical Committee of the Affiliated Traditional Chinese Medicine Hospital of Southwest Medical University.

Inclusion criteria of cases: (1) Patients diagnosed and treated from January 2018 to January 2023; (2) Age ≥ 18 years, ≤ 40 years; (3) The injury of ATFL was diagnosed by medical history, physical examination and imaging examination.

Exclusion criteria of cases: (1) Patients with other organ injuries or limb fractures; (2) Patients with injury of other ligaments of the same side ankle joint; (3) Patients with ankle deformity, disease, or repeated ankle pain and swelling before the injury.

Publicly available single-cell RNA datasets of cartilage tissues, obtained from 5 volunteers. None of the 5 volunteers were diagnosed with ankle-related conditions.

### Instruments

**Fig. 5:**
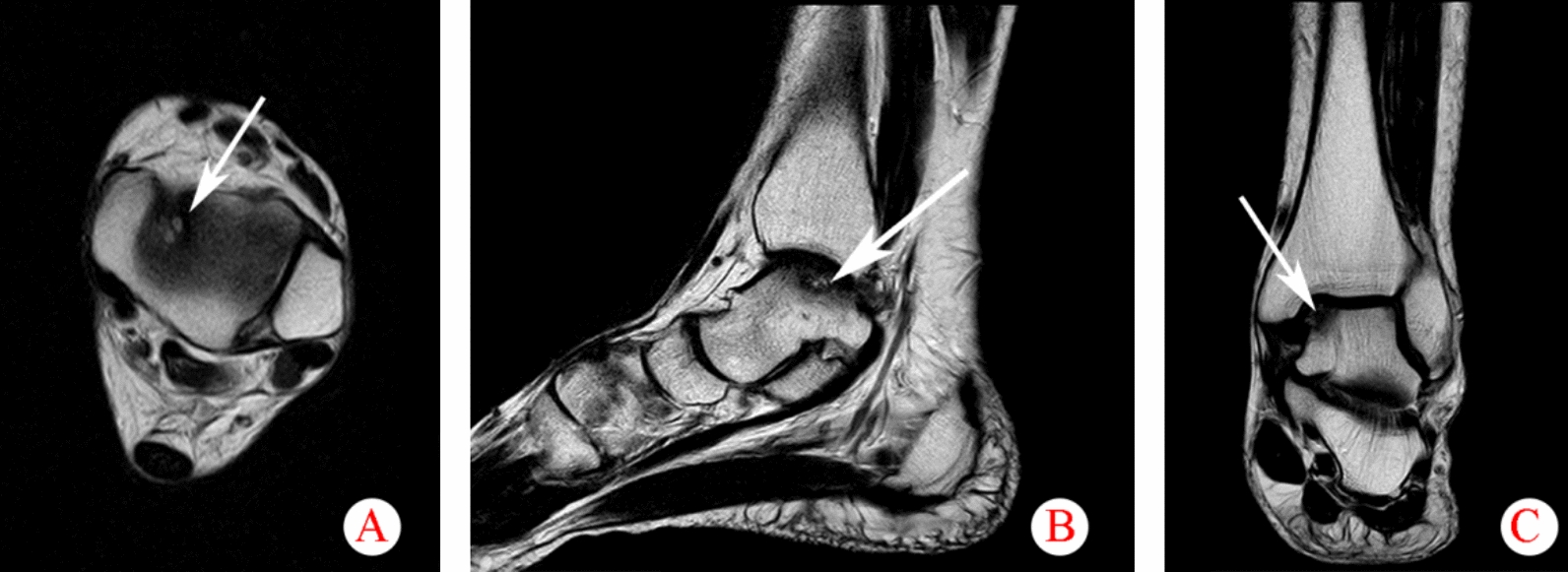
Presentation of osteochondral lesions of the talus in axial, coronal, and sagittal positions. **A** axial position; **B** sagittal position; **C** coronal position. Arrows indicate osteochondral lesions of the talus

All 164 cases of patients with ATFL injury received MRI examination, using PhilipsIntera 1.5 T NovaDual double gradient superconducting MRI scanner, ankle coil, and conventional TSE sequence axial, sagittal, coronal and sagittal SPIR sequence scanning (Fig. 5 A, B and C). Matrix 256 × 256, FOV 14 cm, layer thickness 3 mm. The patient lay supinely, with legs naturally straightened, the transverse axis of the joint was vertical to the axis of the bed length, and the scanning range was upper covering the lower tibiofibular joint, and lower covering the margo inferior calcaneus.

The MRI data obtained were read by two senior physicians of our department, and the degree of damage of the ATFL injury and OLT was diagnosed and measured on the PACS system. In case of disagreement, it shall be decided by superior experts.

Single-cell RNA datasets of healthy talus cartilage obtained from 5 volunteers were processed and integrated by Seurat and Harmony R packages. Cell-Chat was used for analysis of cell–cell communication. Compute-Commun-Prob was used to calculate the communication probability between various cells and infer the communication network between cells. Compute-Commun-Prob-Pathway was used to infer intercellular communication at the signaling pathway level.

### Observation target

#### The classification of ATFL injury and OLT

The classification of MRI findings of ATFL injury was: 0 normal; Grade 1: slight thickening or thinning; Grade 2: partial tear, with increased signal; Grade 3: complete tear, ligament discontinuity or defect, irregular ligament shape and high signal; Grade 4: ambiguous.

The MRI manifestations of OLT were recorded by Hepple classification as follows: stage I: only the surface of articular cartilage was damaged; stage IIa: not only articular cartilage injuries, but also subchondral fractures and peripheral bone marrow edema; Stage IIb: articular cartilage injury and subchondral fracture, but without peripheral bone marrow edema; stage III: osteochondral fragments were separated, but there was no displacement; stage IV: bone fragments were separated, and displaced; stage V: subchondral cysts were formed.

#### Lesion location (the nine equal surface area zones)

Lesion location was determined using a 9-zone anatomic localization scheme on MRI [9].

#### Lesion size (cm^2^)

Measure the long and minor axes of the lesion location, and calculate the lesion size according to the elliptical area calculation formula, π take 3.14. If there are multiple talar cartilage injuries, add them together. Less than 2cm^2^ is a small area of injure, and more than 2cm^2^ is a large area of injury.

#### Thickness index

Thickness index refers to the measurement method in Tao’s article [10].

#### AOFAS

The function of foot was evaluated using the American Orthopaedic Foot and Ankle Association (AOFAS) scoring scale. The result is obtained by simply adding up the scores of each section. The total score is 100 points.

#### VAS

The pain of foot was assessed using Visual Analog Scoring (VAS). Patients were asked to draw a mark on the 10 cm horizontal line based on their own feelings to indicate how pain they suffered.The number from 0 to 10 indicating no pain to severe pain.

### Statistical analysis

All statistical analysis was conducted using SPSS Version 26.0 (IBM, Armonk, NY, USA) with any necessary extensions. The correlation analysis between ATFL injury grade and the Hepple stage of OLT determined by MRI imaging was performed. The chi-square test was used to determine the risk factors associated with OLT by taking gender, age, course of disease, injured side, injured area, and thickness index as independent variables while taking the incidence rate and the severity of OLT as the dependent variable. Multivariate logistic regression analysis was used to determine the independent risk factors, the corresponding odds ratio (OR) and the 95% confidence interval (CI). Respectively, the correlation between ATFL injury grade and the Hepple stage of OLT and AOFAS and VAS were analyzed.

The parameters were expressed as the mean ± standard deviation (SD). All hypothesis tests were performed at a significance level of 5%, with P-values of 0.05 or less considered statistically significant.

## Data Availability

The single-cell RNA sequence datasets collected in the study are presented publicly in the GEO repository, Accession Number GSE216578.
